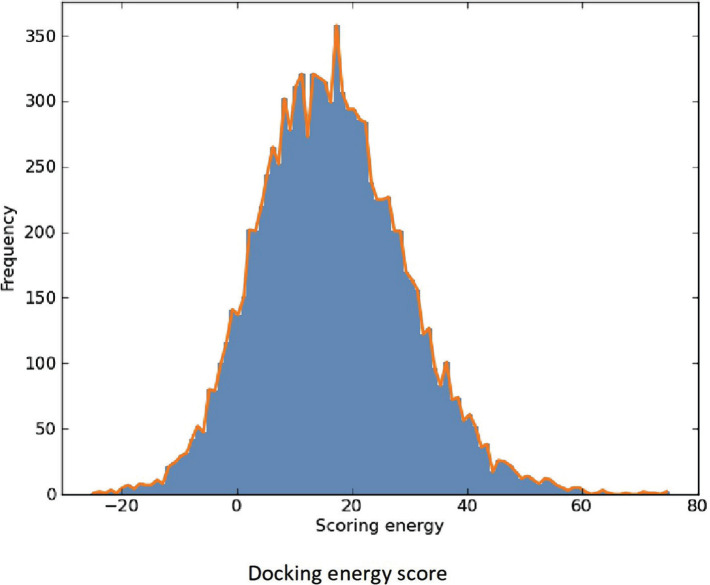# A new variant of the human α-lactalbumin-oleic acid complex as an anticancer agent for chronic myeloid leukemia

**DOI:** 10.25122/jml-2021-0065

**Published:** 2021

**Authors:** Vivek Singh, Ranjana Singh, Dinesh Kumar, Abbas Ali Mahdi, Anil Kumar Tripathi

**Affiliations:** 1. Department of Biochemistry, King George’s Medical University, Lucknow, Uttar Pradesh, India; 2. Centre of Biomedical Research, Sanjay Gandhi Post-Graduate Institute of Medical Sciences, Lucknow, Uttar Pradesh, India; 3. Department of Clinical Hematology, King George’s Medical University, Lucknow, Uttar Pradesh, India

**Keywords:** Alpha-lactalbumin, Oleic acid, Chronic myeloid leukemia, SNARE, IL-8, Survivin, HALOA – Human α-Lactalbumin-Oleic Acid, EDTA – Ethylenediaminetetraacetic acid, ICP-OES – Inductively coupled plasma-optical emission spectrometry, UV-CD – Ultraviolet-Circular Dichroism spectroscopy, SDS-PAGE – Sodium dodecyl sulfate polyacrylamide gel electrophoresis, OA – Oleic acid, ANS – 1-anilino-8-naphthalene sulfonate, NMR – Nuclear Magnetic Resonance, TEM – transmission electron microscopy, IL-8 – Interleukin 8, SNARE’s – SNAP Receptor, CML – Chronic myeloid leukemia, MTT – 3-(4,5-dimethylthiazol-2-yl)-2,5-diphenyl tetrazolium bromide

## Abstract

Chronic myeloid leukemia (CML) is a clonal myeloproliferative disorder of hematopoietic stem cells. Although there have been advancements in treatment, there is still a need to develop a biotherapeutic agent. A new variant of the human alpha-lactalbumin-oleic acid (HALOA) complex has been synthesized, which showed similarities with SNARE. The native α-LA was treated with EDTA to remove Ca^2+^ ions confirmed by ICP-OES and Arsenazo III to unfold and attain apo structure. The apo LA was mixed with OA in a specific ratio, leading to HALOA complex formation. The conformational state from native to complex was elucidated by circular dichroism (far; 190–260 nm and near; 260–340 nm UV-CD), which confirmed that the complex consists of a majority of turns and β-sheet structure. SDS-PAGE result showed the masking effect of OA on apo α-LA. In the lane of the complex, there was no band detected. However, 1-anilino-8-naphthalene sulfonate (ANS) dye has shown maximum fluorescence intensity with the complex because of the availability of hydrophobic patches, which was further validated by NMR spectroscopy indicating the masking effect of OA on the apo α-LA. The SNARE behavior of the complex (500 nm) has been confirmed by TEM. This new structural variant complex shows anti-tumor activity on chronic myeloid leukemia by targeting the IL-8, survivin, and induces apoptosis through DNA fragmentation, but not against normal cells. Overall, the formulated complex shows that SNARE-like behavior can be used as a promising anti-tumor agent with lower toxicity and maximum bioavailability.

## Introduction

Human alpha-Lactalbumin (α-LA) is a calcium-binding protein (14.2 kDa), which has neutraceutical values [1]. A new paradigm focuses on its capacity to interact with oleic acid (OA), forming a protein-lipid complex with a property to trigger apoptosis in tumor cells [2–9]. Some studies have already reported that altering the temperature and pH of α-LA attains the molten globule (MG) conformation upon binding with OA [10–17]. Several studies originated and inspired by the Svanborg laboratory mention that this active complex was developed by chromatographic procedures, not by mixing two components [18]. However, few studies [18, 19] have inferred that the mixing process could produce a complex at lower pH conditions that display conformation and biological properties similar to the complex formed by Svanborg through chromatographic procedures.

Chronic myeloid leukemia (CML) is a clonal myeloproliferative disorder. The cure rates of CML have improved, but resistance to drugs leads to recurrence, causing death in 50% to 95% of patients. In addition, CML patients frequently suffer from secondary neoplasms and chronic health-related problems [20]. Survivin (inhibitor of apoptosis) plays a crucial role in cell proliferation and apoptosis [21, 22]. Increased expression of survivin leads to hematopoietic malignancies [23]. Previous studies indicated that the anti-apoptotic marker survivin tightly regulates most leukemia cells present in the G2/M phase. Survivin interacts with the non-homologous end joining (NHEJ) DNA repair complex that maintains the integrity of the genome in cancer cells and leads to inflammation (IL-8 production) [24–26]. IL-8 represents one of the neutrophil chemotactic activating factors. IL-8 production is induced by oxidative stress, while antioxidant therapy inhibits the IL-8 level [27–30]. To our knowledge, the complex is formed by mixing apo α-LA with OA at neutral pH (7.4). However, structural similarity with SNARE’s complex has not been reported. We aimed to (1) make a new variant of the protein-lipid complex at neutral pH for CML treatment and (2) elucidate the effect of the HALOA complex on the markers of apoptosis (total antioxidant capacity, IL-8, and survivin) on leukemia cells.

## Material and Methods

Materials used in this study are Human α-lactalbumin (Sigma), oleic acid (Sigma), G25 Sephadex (Sigma), ANS (Sigma), RPMI-1640 media (Gibco), antimycotic and antibacterial (Thermo fisher), FBS (Gibco), MTT assay (ab211091 MTT Cell Proliferation Assay Kit), Trypan blue (Thermo), Giemsa (Sigma), Hoechst dye (ab228551 Hoechst 33342), DNA Fragmentation kit (TaKaRa 6137) TAC kit (sigma), Annexin V-FITC assay (Milentyi), EdU Assay (Abcam), Survivin assay (Abcam).

### HALOA complex formation

First, 25 milligrams of native human α-LA were solubilized in 1.8 mM in Tris (10 mM Tris. Cl at pH 7.4) at room temperature. Furthermore, we added 3.5 mM EDTA, which helped remove bound Ca^2+^ from native α-LA and pass through a gel filtration column (G-25 Sephadex column). After proper elution with Tris buffer at pH 7.4, we obtained an apostate of α-LA. The concentration of α-LA (native, apo, and complex) was measured using the Nanodrop at 280 nm wavelengths, and apo α-LA without calcium ion was lyophilized. Following this, apo α-LA was dissolved in DPBS at pH 7.4 during dissolution; the protein sample was incubated at 37°C for 10 minutes to maintain apo-form integrity at pH 7.4. We obtained 5 mM oleic acid stock solution by blending OA solution in chloroform - DOPS (1, 2-dioleoyl phosphatidylserine) and DOPC (1, 2-dioleoyl phosphatidylcholine); in 30:70 mol/mol ratios in falcon tube (15 mL). OA solution was dried with a gentle shower of nitrogen and resuspended in DPBS at a pH of 7.4. HALOA complex was formulated by simply mixing apo α-LA (1 mg/mL) with OA (1 mg/mL) in a 1:1 ratio at pH 7.4, vortexing for 10 min at 37°C for better dissolution. The absorbance of the complex was taken at 200–900 nm at a different concentration, and further validated by different biophysical techniques, as explained below.

### Circular dichroism (CD) for structural characterization

CD spectrum was procured by JASCO J-1500 spectropolarimeter. Quartz cuvettes with 10 mm path length were used, and a CD spectrum was recorded from wavelength 190 nm to 320 nm. The wavelength step was 1 nm, the scan rate was 10 nm/min, and the response time was 4s. A total of three scans were recorded and taken on average for each spectrum. In addition, baseline spectra were recorded with DPBS in each cuvette and subtracted from the CD spectra of native α-LA, apo α-LA, and HALOA complex.

### ANS fluorescence spectroscopy for confirmation of exposed hydrophobic patches on protein

ANS fluorescence emission spectra were procured at 25°C on a Perkin–Elmer spectrometer using a quartz cuvette with a 1 cm excitation path length, from 400 nm to 600 nm wavelength range excitation wavelength at 385 nm. Both the excitation and emission bandpass were set to 5 nm. Stock solutions were prepared by dissolving native α-LA, apo α-LA, and HALOA complex in 10 mM potassium phosphate buffer at pH 7.4. The concentrations were depicted by amino acid analysis after acid hydrolysis, and spectra were recorded on aliquots diluted in 10 mM potassium phosphate buffer at pH 7.4.

### SDS-PAGE for the integrity of the protein

Native α-LA, apo α-LA, and HALOA complex were separated using SDS-PAGE. Then, we prepared 4–5% stacking and 12% resolving gels and subjected them to electrophoresis following the standard protocol [31].

### Inductively coupled plasma optical emission spectrometry (ICP-OES) for metal (Ca^2+^) detection.

Calcium ion was analyzed from protein samples (Native, apo alpha-lactalbumin, and HALOA complex) diluted in a 1:3 ratio. Multi-element standard (Perkin Elmer pure plus, USA) stock solution 1000 mg/L was used to prepare calibration standard solution at a different concentration at 0.005 to 1 mg/L. The clear solution obtained after acid digestion was kept in precleaned tubes after cooling. The sample was analyzed by using ICP-OES (Optima 8000, Perkin Elmer) at three different time periods. Instrumental conditions for calcium analysis are given in Table 1.

**Table 1.** Operating conditions for ICP-OES.

**No.**	**Instrumental Conditions**	**Value**
**1.**	Plasma Gas Flow (L/min)	8
**2.**	Auxiliary Gas Flow (L/min)	0.2
**3.**	Carrier Gas Flow (L/min)	0.55
**4.**	RF Power (W)	1300
**5.**	Plasma View	Axial
**6.**	Sample Flow Rate (ml/min)	1.0

### Arsenazo-III used for Calcium ion removal from protein

We added 20 μL of native α-LA, apo α-LA, and HALOA complex to 2 ml of reagent consisting of boric acid-KC1- NaOH buffer, 50 mmol/L, pH 7.4, to contain per liter. Arsenazo-III, 0.08 g 8HQS, 1.13 g, Triton X-100, 0.5 gram without the clearing factors were incubated at room temperature for 2 min. The absorbance was measured at 650 nm against a blank [32].

### ^1^H NMR Spectra confirms the masking effect of OA on Apo α-LA by titration method

The spectra of ^1^H NMR were recorded using a Bruker spectrometer at 900 MHz in D_2_O. Lyophilized HALOA complex, native, and Apo α-LA (2.5 mg) was dissolved in 100 μL of D_2_O, and the pH was maintained at 7.0 using NaOD. Oleic acid (4 mg) was dissolved in 75 μL ethanol, and 10 μL were added to 250 μL D_2_O. Apo α-LA for ^1^H NMR was generated by dissolving α-LA in double-distilled water (deuterated) containing 10-fold molar excess of EDTA at pH 8.0. All the spectra were recorded at 900 MHz.

### Transmission electron microscope (TEM) for the confirmation of HALOA like SNARE’s

A small drop of HALOA complex (usually about 4 μL) was pipetted onto a formvar-coated copper grid and allowed to dry at room temperature. Complex analysis was performed under FEI Tecnai G2 spirit twin transmission electron microscope equipped with Gatan digital CCD camera (Netherland) at 80KV.

### Cell culture

Cells (NIH and K562 cell line) were maintained in a culture medium composed of RPMI-1640, fetal bovine serum (5%), antimycotic, and antibacterial (0.1%) in 50 mL of stock solution. Cell viability was checked by MTT assay (cells were inoculated at 5,000 cells/well on 96-well plates and incubated for 24 to 72 hours before the HALOA complex treatment). We continued cell treatments with HALOA complex (1 mg/mL, 0.5 mg/mL, 0.25 mg/mL, 0.125 mg/mL), Apo α-LA (1 mg/mL), and OA (1 mg/mL) for 24 hrs. to 72 hrs. 20 μL aliquot of 3-(4, 5-Dimethylthiazol-2-yl)-2,5 diphenyltetraoliumbromide (MTT, a yellow tetrazole; in PBS) was added to the wells, then incubated in a CO_2_ incubator for two hours at 37°C. After removing the supernatant carefully, 200 μL of DMSO was added and mixed, and the absorbance was recorded at 563 nm.

### DNA damaging detected during the treatment by HALOA complex

DNA fragmentation was detected using agarose gel electrophoresis as per manufacturer guidelines. The cell suspension remaining after Trypan blue (970 μL, 2 × 10^6^/mL) was lysed in 5 mM Tris, 20 mM EDTA, 0.5% Triton X-100 (pH 8.0) at 4°C for 1 hour and centrifuged at 13,000 rpm for 15 min. DNA was precipitated in ethanol overnight at −20°C and further treated with proteinase K and RNAse. Furthermore, we loaded 1.0% agarose gels into the electrophoresis unit, applying a constant voltage of 70 V for 2 hours. DNA fragments were visualized with ethidium bromide using the Bio-Rad gel doc.

### Apoptosis by Annexin V-FITC assay

For apoptosis analysis, cells were seeded in 6-well plates at a density of 2.0 × 10^5^ cells/well and allowed to grow overnight. The next day, the cells were treated with HALOA (0.5 mg/mL) for 24 h at 37°C. Untreated cells were used as control. After incubation, the cells were harvested and washed twice with cold PBS. Next, 1×10^5^ cells were dispersed in 100 μL of 1 × Annexin V binding buffer. Subsequently, 5 μL of Annexin V- FITC and 5 μL of PI and PE Texas red were added, and cells were incubated at room temperature in the dark for 15 min. Finally, 400 μL of 1 × Annexin V binding buffer was added under gentle mixing, and the samples were analyzed using flow cytometry (BD Biosciences, San Jose, CA) and FlowJo software.

### Cell proliferation by EdU assay

Cell proliferation and DNA synthesis were determined using a Click-iT® EdUiFluor® 488 Assay Kit (ab219801) according to the manufacturer’s protocol regarding FACS and fluorescence microscopy.

### Apoptosis Markers Assay

ELISA was used to check the expression level of survivin (ab119607- Survivin Human ELISA Kit) and IL8 (Ray Bio® Human IL-8 ELISA Kit) in cell lysate. The colorimetric assay was used to confirm the total antioxidant (TAC) of cells after treatment with HALOA complex using an antioxidant assay kit (Sigma Aldrich- Catalog Number CS0790), following the manufacturer’s protocol. Bioinformatics tools used for docking were Swiss dock and Pydock.

### Statistical analysis

All the data were expressed as mean ±standard deviation (SD). The results were analyzed using SPSS 21.0 version and Graph pad software (Prism 5). The significance of differences was analyzed using one-way ANOVA and Student’s t-test (*p <0.05).

## Results

We used Human alpha-lactalbumin as starting material, converted it into the HALOA complex, and then validated its anti-tumor activity on the K562 cell line and NIH (normal cell line).

### In-silico study reveals the interaction of apo α-LA with OA

**Figure 1.**
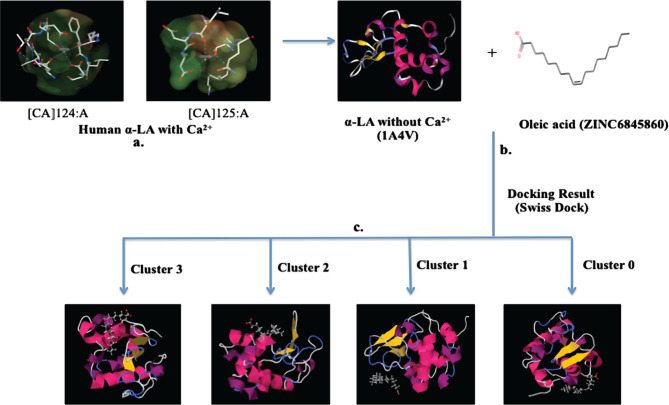
Interaction of oleate with Human α-LA. (a, b) Removal of calcium ions from human α-LA leads to formulation of HALOA complex. (c) Furthermore, docking was performed with oleate by using Swiss dock tools.

Formulation of the HALOA complex was done by removing calcium ions from human α-LA (Figure 1 a, b). Furthermore, docking was performed with oleic acid using Swiss dock tools, as shown in Figure 1 c. We found 46 clusters with different binding energy efficiency. The result indicated that oleic acid resides around apo α-LA but is not found in the pocket of calcium ion (the free energy table is attached as supplementary data).

### Formation and structural characterization of HALOA Complex

**Figure 2.**
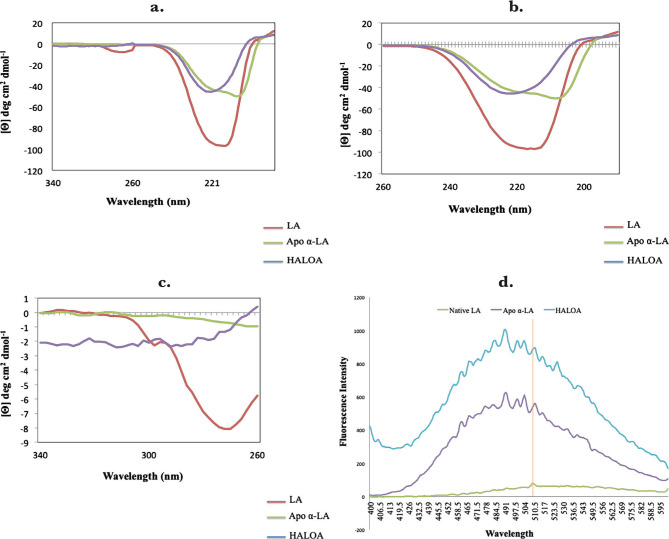

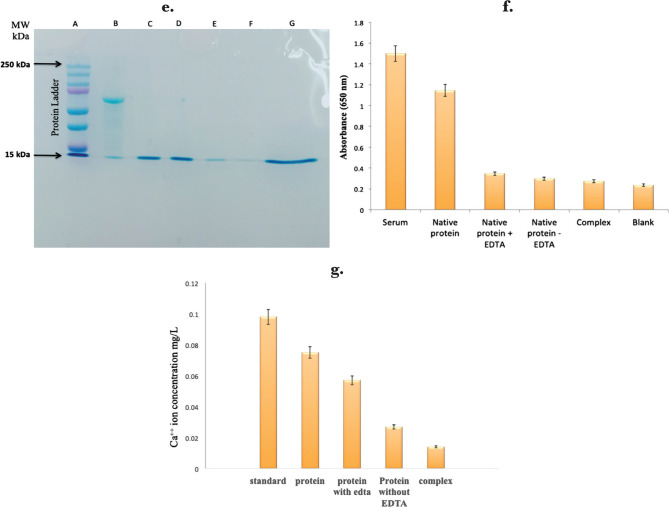
HALOA complex validation; (a) combined graph of Near and Far UV-CD spectra, red color shows Human lactalbumin (LA) which contain turns, the green color shows the Apo α-LA, whereas, the violet indicates the HALOA complex consisting of a beta-sheet and turns. (b, c) far (190–260 nm) and near (260–340 nm) UV-CD Spectra shows a loss in the native structure of α-LA during the conversion into HALOA. (d) ANS fluorescence spectra of Native LA (green color), Apo α-LA (violet color), and HALOA (sky blue) show the hypsochromic shift towards the lower wavelength. (e) SDS-PAGE result in which lane C, G shows native alpha-lactalbumin with calcium ion; lane D, E shows without calcium ion, lane F for complex but no band found in this lane because of masking effect of oleic acid on Apo α-LA, lane A for protein ladder and B shows BSA. (f) ICP result used to determine the calcium ion concentration. The concentration of calcium ion was found to be high in native human α-LA compared to EDTA treated LA, and the result of the complex, with and without EDTA, shows the value around the blank (no calcium ion). (g) Arsenazo III binds with the calcium ion; its absorbance is highest in the native protein compared to EDTA, treated protein, and complex form of protein. In contrast, the result was approximate as a negative control, which indicated that the complex does not have calcium ions.

The UV absorbance spectrum was recorded to compare the structure of the native and apo forms of α-LA at room temperature. We found that apo α-LA isolated by gel filtration is not similar to the native form of LA shown in Figure 2 a–c. The CD data were analyzed using BEST SEL web service tools, and we observed that the structure of native LA contains the majority of turns. Simultaneously, the HALOA complex constitutes 17.3% anti-parallel beta-sheet and 82.7% turn (data attached in a supplementary form). HALOA complex and LA (apo and native) show an increase in CD amplitude around the 200–230 nm region. The trajectory of CD spectra of native LA and complex are the same. The near UV-CD region demonstrates that both the complex and the LA show a marked reduction in negative ellipticity around the 260–300 nm wavelength region. There is a peculiar loss in signal trajectory around tyrosine and tryptophan regions, as shown in Figure 2 c. Overall, these observations indicate an alteration in the globular structure from the native state to the formulated HALOA complex, as shown in Figure 2.

The exposed hydrophobic amino acid cluster of protein was widely detected through binding with fluorescent probe ANS, which significantly increases fluorescence intensity at emission wavelengths from 510 to 480 nm. Native LA failed to bind ANS because of the unavailability of hydrophobic parts, as shown in Figure 2 d, with low fluorescence intensity at 510 nm. In contrast, the apo α-LA showed enhanced intensity at wavelength 477 nm, whereas the HALOA complex showed maximum fluorescence at 488 nm due to the absence of Ca^2+^ ions. Consequently, we can say that the apo α-LA and HALOA complex have a high affinity to bind with ANS dye than the native form of α-LA because of the availability of hydrophobic patches. We have observed a hypsochromic shift in the graph towards a lower wavelength. The masking effect of OA was validated by SDS-PAGE, and we observed that LA integrity did not alter lanes C, D, E, G (Figure 2 e). There was no band found in complex lane F. This result indicates the aggregation behavior of oleic acid around apo α-LA, which was further validated and confirmed by NMR and TEM techniques. ICP-OES and Arsenazo-III confirmed removing calcium ions (Figure 2 f ); figure 2 g is showing that the concentration of calcium ion 0.018 mg/L is negligible in an account of standard Whey Protein and Native alpha-lactalbumin, while Arsenazo-III dye binds with available calcium ion in the solution of unfolded protein.

### NMR and TEM confirm the HALOA complex attains a structure as SNARE’s

**Figure 3.**
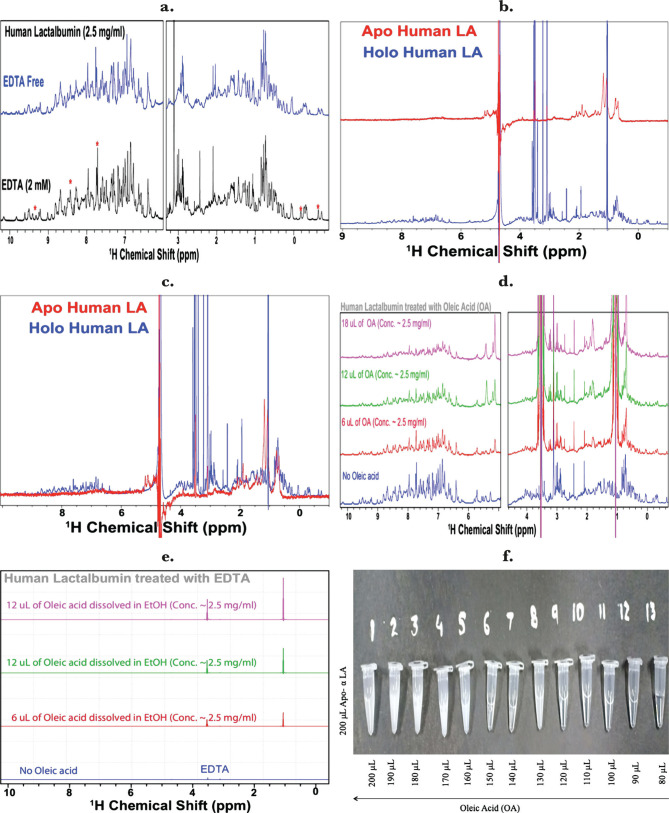

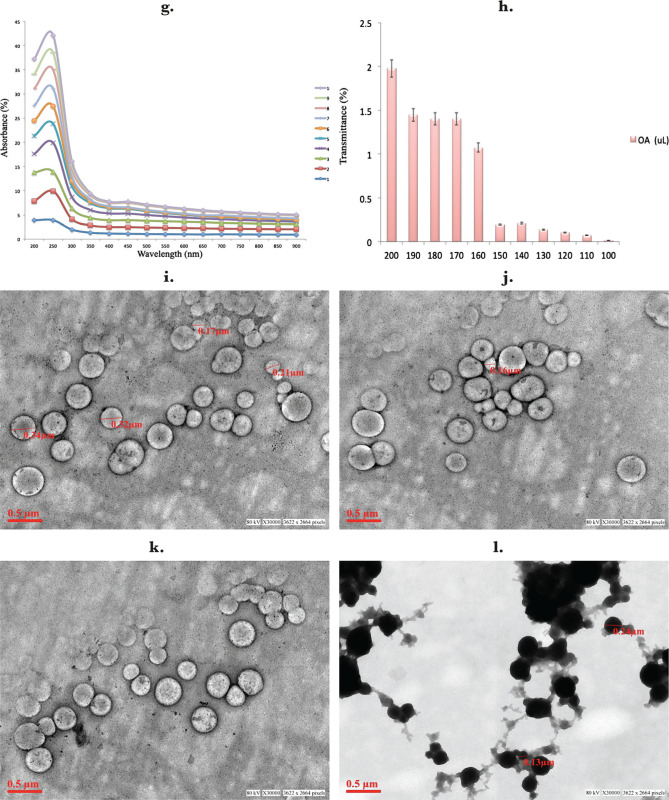
HALOA complex attains the similarity with SNARE's complex; (a) 2.5 mg of native LA was dissolved in DPBS. We recorded the spectra at 900 MHz and, when added to EDTA (2 mM), we found the loss of Apo α-LA in spectra, marked with a red asterisk. (b, c) NMR spectral pattern of apo and holo (native LA) forms of human α-LA was different. Compared to the holo form, the NMR signals of the apo form of Human LA are significantly reduced, suggesting that the protein is possibly forming the apostate in the absence of Ca^2+^ ions. (d, e) NMR signals of apo α-LA progressively decrease upon titration with Oleic acid, suggesting a strong interaction between protein and Oleic acid. The decreased intensity of the NMR signal upon the addition of oleic acid suggested that the HALOA complex is not contributing to the ^1^H NMR signal. Characterization of changes in aggregation state OA after the addition of Apo α-LA. (f, g) turbidity measurement and absorbance of this titration taken from 200–900 nm wavelength. (h) Turbiditimetric analysis; Addition of OA solutions (200 μL- 100 μL) in Apo α-LA (200 μL). The absorbance of solutions was recorded at 400 nm. (i–l) size of HALOA complex measured by TEM in bright and darkfield, which shows a 500 nm range of size that is the exact size of t-SNARE's complex.

The spectra of ^1^H NMR (900 MHz) signal of native α-LA shows characteristics of a proper folded globular structure indicated by narrow lines and a significant shift in the dispersion of many strong signals seen around the aromatic region (around 7 ppm), with a frequent shift in methyl signal (between 0-1ppm) as shown in Figure 3 a. However, the NMR spectral pattern of the apo form of LA was different in Figure 3 b, c, compared to the native form. The NMR signals of apo were significantly reduced, suggesting that the protein could attain the apostate in the absence of Ca^2+^. The NMR signals of apo α-LA significantly decreased upon titration with OA, suggesting a strong interaction between apo α-LA and OA (Figure 3 d, e). We found that by increasing the volume of OA (6 μL, 12 μL, and 18 μL), there is a decrease in the intensity of NMR signals.

The TEM result confirmed that the encapsulation of apo α-LA within OA forms a product similar to SNARE’s complex. Previous literature indicates that OA could attain the structure of micelles at pH more than 10.5, and micelles (single layered) further form plasma membrane like vesicles (double layered) if the pH is decreased below 9.0 [33–35]. Turbidity measurements demonstrate surface hydrophobicity by increasing the concentration of OA in an aqueous solution of apo α-LA at different wavelengths, as shown in Figure 3 f–h. The result shown in Figure 3 i–l reveals that the overall size of complex formed by adding OA with apo α-LA is within the range of 500 nm, like SNARE’s complex (Figure 3). Therefore, SNARE’s like behavior of the formulated complex is analogous to similar effects elucidated when proteins like tau [36], alpha-synuclein [37] were added in solutions of fatty acid or an anionic detergent.

### Cell culture and cell viability assay

**Figure 4.**
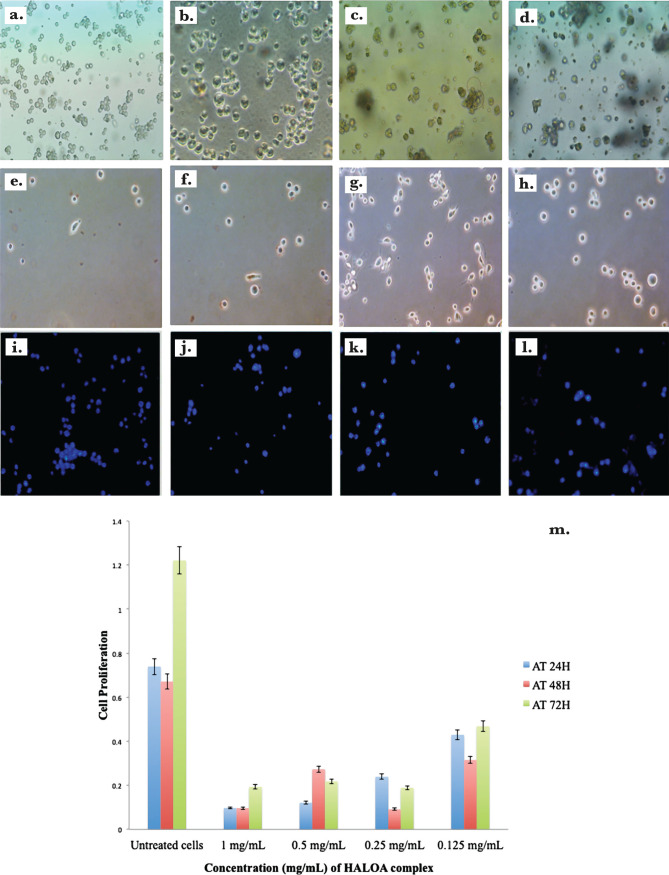
K562 Chronic myeloid leukemia cell lines, (b) Apo α-LA (1 mg/ml) treated cells, (c) OA acid (1 mg/mL) treated cells show apoptosis in K562 cells (d–h) cells treated with different conc. of the HALOA complex (d. 2 mg/mL, e. 1 mg/mL, f. 0.5 mg/mL, g. 0.25 mg/mL, h. 0.125 mg/mL). (i–l) Hoechst-stained dye shows a low intensity using treatment with HALOA complex (i. K562 cells, j. 1 mg/mL, k. 0.5 mg/mL, l. 0.25 mg/mL). (m) cell viability assessed by MTT assay, from where we calculated the IC_50_ value by which we decided the dose of HALOA complex (0.5 mg/mL) that was further used for the study.

NIH (normal cell line for toxicity check of complex) and K562 cell lines (chronic myeloid leukemia) were treated with LA (Apo α-LA; 1 mg/mL), OA (1 mg/mL), and HALOA complex (1 mg/mL, 0.5 mg/mL, 0.25 mg/mL, 0.125 mg/mL). Cell viability was determined at 24, 48, and 72 hrs. after treatment with formulated candidates by MTT reagent, following the manufacturer’s protocol. From this result, we calculated the IC_50_ which indicates that 0.5 mg/ml of HALOA complex shows the best result without any cell cytotoxicity as shown in Figure 4. The IC_50_ value is the most effective at a concentration of 0.5 mg/mL, which was further used throughout this study.

### Biological activity of HALOA complex and its effect on molecular markers (TAC, IL-8, and Survivin)

**Figure 5.**
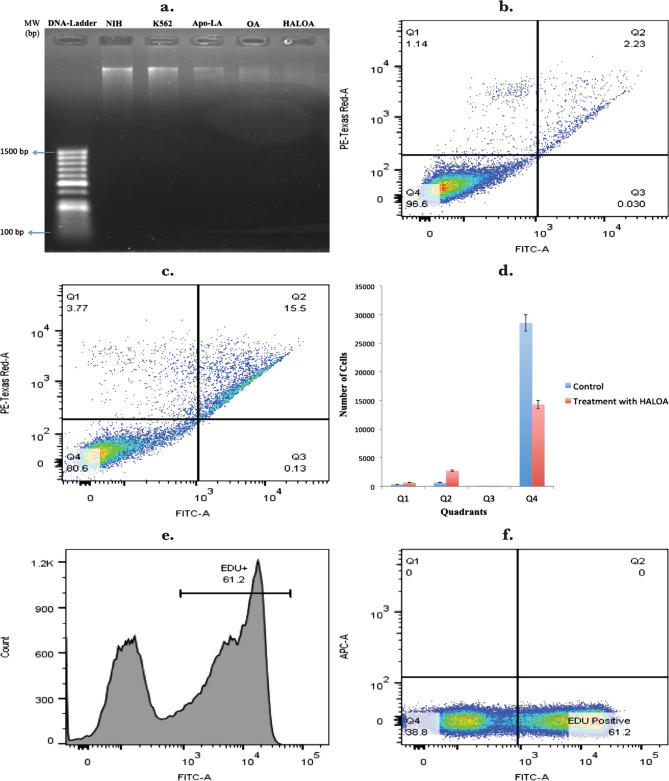

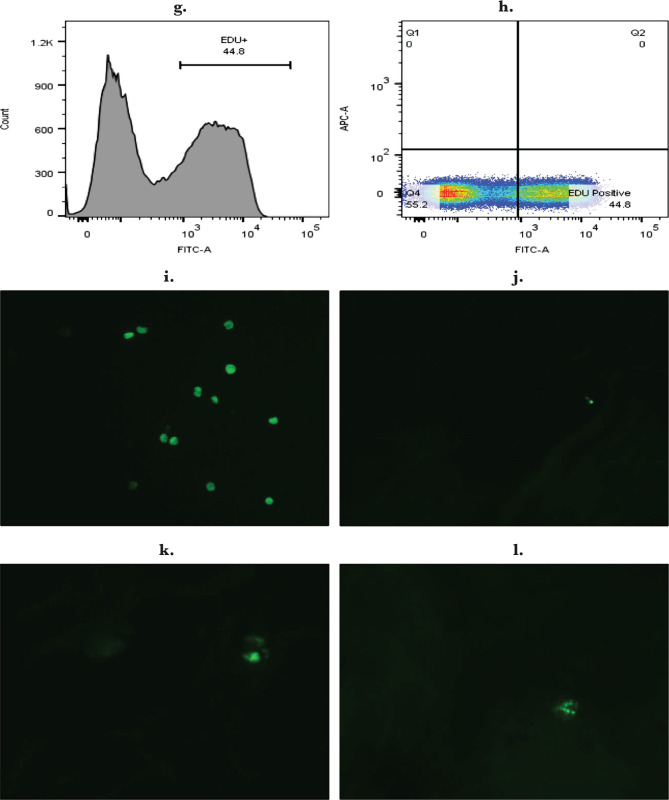

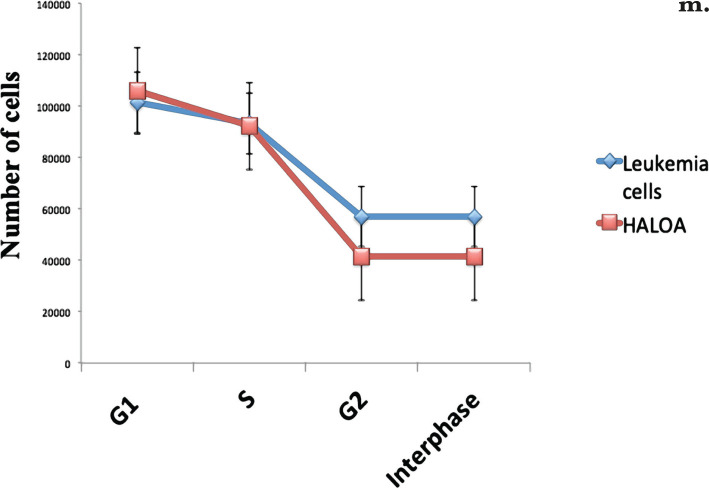
Complex leads apoptosis in K562 cells; (a) DNA fragmentation analyzed on normal cell line (NIH). In the first column, we found no DNA damage by HALOA complex (0.5 mg/mL) compared with DNA of K562 cell line, but in HALOA (0.5 mg/mL), Apo α-LA (1 mg/mL), and OA (1 mg/mL) the column found damaged DNA. (b–d) Phosphatidylserine externalization is a lead marker for apoptosis which was confirmed by FACS (Annexin V-FITC assay) (c) K562 cells as the control without treatment in which Q1- Necrotic cells, Q2- Late apoptotic, Q3- Early apoptotic cells, and Q4-live cells found statistically significant results with a p-value of 0.012. This experiment was performed within 8 h, which shows the significantly increase in no. of apoptotic cells by HALOA (0.5 mg/mL) complex as shown in (c). (d) bar diagram decreased in the cell in different quadrants with a p-value of 0.012. Cell proliferation assay by EdU dye (e–m) shows a 16.4-fold decrease in the G2/M population by the HALOA complex (0.5 mg/mL). (e–h) shows a reduction in cell proliferation rate (73.20%) by the HALOA complex (0.5 mg/mL). (i–l) shows the fluorescence of EdU dye under a fluorescence microscope, which indicated that a decrease in the population of live cells (fig. i) after treatment with HALOA (0.5 mg/mL) (J), Apo α-LA (1 mg/mL) (k), and OA (1 mg/mL) (l) shows a significant reduction in cells within 72h and (m) shows a reduction in cell population maximum in the G2/M phase by the treatment with complex (0.5 mg/mL).

The HALOA complex (0.5 mg/mL), Apo α-LA (1 mg/mL), and OA (1 mg/mL) significantly reduced cell viability in 24 hours. DNA fragmentation was observed 8 hours after the HALOA complex treated K562 cells, but no DNA fragmentation was found in NIH cells, as shown in Figure 5 a. To determine whether the growth inhibition of HALOA complex (0.5 mg/mL) in K562 cells was associated with programmed cell death, apoptosis rate was evaluated using AnnexinV-FITC staining. K562 cells were evaluated before and after treatment with HALOA complex by flow cytometry within 24 hours, showing the death index rate by 57.1%. Result obtained from FACS was analyzed using FlowJo software. Quadrant Q1 showed necrotic cells, Q2 late apoptotic cells, Q3 early apoptotic cells, and Q4 showed live cells (Figure 5 b–d). The result was obtained using a t-test with a p-value of 0.012. K562 cells were grown to 80–90% confluence in 6 well plates and analyzed by FACS and fluorescence microscope. After the treatment with HALOA complex (0.5 mg/mL), we explored the DNA content of K562 cells during the cell cycle progression using EdU. We found that the complex inhibits maximum growth in the G2/M phase of K562 cells, reducing cell proliferation rate by 73.20% and fold change of 16.4 (Figure 5 e–m). Under the fluorescence microscope, there is a significant reduction in fluorescence intensity within 72 hrs.

**Figure 6.**
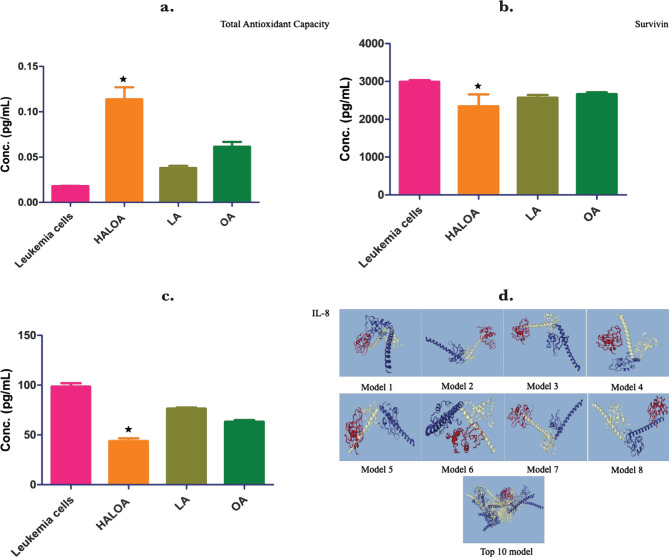
Effect of complex on TAC, survivin & IL-8; (a) total antioxidant (TAC) was found to be significantly increased in HALOA (0.5 mg/mL) treated cells (0.113±0.013 mM, p-value; 0.0064) and followed by LA (Apo α-LA (1 mg/mL), OA (1 mg/mL) (0.038±0.002 mM, p-value; 0.0061, 0.061±0.005 mM, p-value; 0.051) as compared with leukemia cells (0.018±0.0003 mM). (b) the complex lowers down the expression of survivin as the conc. in leukemia cells (2990±43.13 pg/mL), HALOA (0.5 mg/mL) treated cells (2346±1.12 pg/mL, p-value; 0.006), LA (Apo α-LA (1 mg/mL) (2573±67.88 pg/mL, p-value; 0.027), and OA (1 mg/mL) (2666±45.25 pg/mL, p-value; 0.003). (c) effect of complex on inflammation factor (IL-8) in leukemia cells (98.62±3.36 pg/mL), HALOA (0.5 mg/mL) (43.83±2.75 pg/mL, p-value; 0.05), LA (Apo α-LA (1 mg/mL) (76.59±1.02 pg/mL, p-value; 0.048), and OA (1 mg/mL) (63.31±1.69 pg/mL, p-value; 0.021). (d) the expression level of survivin and their association with HALOA complex, after G2/M cell cycle assessment; the docking result on the site of Thr 34 is a central part of viability after phosphorylation expression level of surviving significantly decreased in the treatment with complex (p=0.034, p=0.023, and p=0.033). All the results are presented in the form of Mean±SD. The graph is plotted with SD (*p<0.05).

HALOA complex increases total antioxidant level in the K562 cell line (0.113±0.013 mM, p-value; 0.006) as shown in Figure 6 a. Survivin was reported as a gatekeeper at the G2/M phase boundary of cell cycles [38]. The expression of survivin was found to be very high in K562 cells (2990±43.13 pg/mL), but after treatment with OA, Apo α-LA and complex (2666±45.25 pg/mL, p-value; 0.003), (LA; 2573±67.88 pg/mL, p-value; 0.027), (2346±1.12 pg/mL, p-value; 0.006) its expression significantly decreased (Figure 6 b). Moreover, we examined the expression level of IL8 and total antioxidant (TAC) and found that the complex significantly reduces IL-8 level (43.83±2.75 pg/mL, p-value; 0.05) as shown in Figure 6 c. The maximum reduction was found in a case of complex treated cells, which might indicate that the complex can target survivin. This result was further validated by the docking (Pydock) method, which indicates that HALOA (red color) has a high affinity to bind at a phosphorylated site (at 34) of survivin (white and blue color), as shown in model 1 (Figure 6 d). We got a total of 10 docking models (from the Pydock server) with different energy scores allowed, as shown in Figure 6 d. All values are presented in Table 2.

**Table 2.** Mean±SD (P-value).

**No.**	**Parameters (Conc.)**	**Control** **(Mean±SD)**	**Apo- α LA** **(Mean±SD)**	**OA** **(Mean±SD)**	**HALOA** **(Mean±SD)**	**P-value**
**1.**	TAC (mM)	0.018±0.0003	0.038±0.002	0.061±0.005	0.113±0.013	0.0061 0.051 0.0064
**2.**	IL-8 (pg/mL)	98.62±3.36	76.59±1.02	63.31±1.69	43.83±2.75	0.048 0.021 0.05
**3.**	Survivin (pg/mL)	2990±43.13	2573±67.88	2666±45.25	2346±1.12	0.027 0.003 0.006

## Discussion

**Figure 7.**
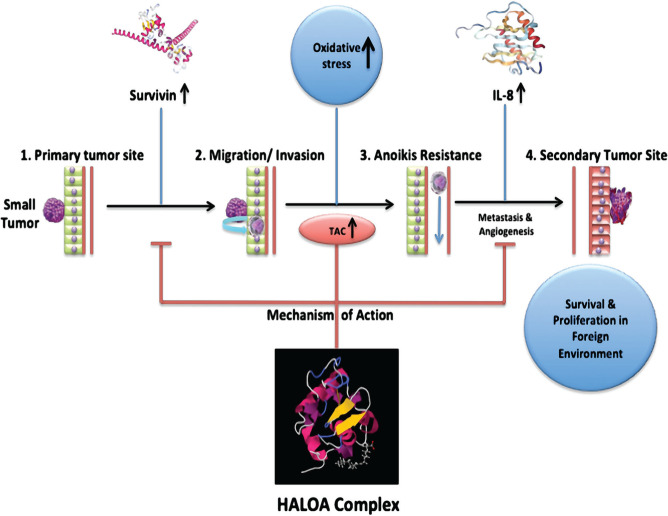
Overall molecular action of the HALOA complex on the combined factors.

Human α-LA can vary its biological function according to its conformational state. Fatty acid (oleic acid C18:1) acts as a cofactor for the protein-lipid complex. Our finding agrees with other studies that explore the formation of a new variant (HALOA complex) by altering the conformational state of alpha-lactalbumin from native to apo state, followed by a mixture with oleic acid. Our result showed that the formed HALOA complex resembles SNARE behavior. EDTA was used to help release calcium ions and induce the unfolding in native α-LA, confirmed by spectroscopic techniques. The calcium ion removal helps accessing the hydrophobic patches in α-LA, which is probed by ANS dye. Moreover, the formulated HALOA complex increases fluorescence intensity and shifts signal towards a lower wavelength (blue shift) when juxtaposed with its native α-LA. The near UV-CD spectroscopy of native α-LA had maximum amplitude at 274 nm because of tyrosine residues and at 296 nm because of tryptophan amino acids. Still, there is a significant loss of signal in the case of the apo α-LA and HALOA complex, which indicates the unfolding of the protein. The NMR spectrum of Apo α-LA progressively decreased upon titration with oleic acid, suggesting that there is a strong interaction between Apo α-LA and OA. However, the TEM result states that the addition of OA helps in the encapsulation of apo α-LA within OA, forming a unilamellar complex that attains size (500 nm), just like SNARE. Several reports documented that the t-SNARE’s and secretory cargo complex or V-SNARE’s are part of the conserved protein domains involved in a coalition of opposing membranes [39]. So the formulated complex with SNARE-like behavior will be helpful in effective targeting without loss of energy. The apoptosis induced by the HALOA complex was investigated based on the quintessential trait of programmed cell death, i.e., cell shrinkage, DNA fragmentation, and phosphatidylserine externalization. Our findings showed shrinkage in cell volume and enhanced granularity after treatment with the HALOA complex on K562 cells, which was not observed in OA and Apo α-LA. HALOA complex induces DNA fragmentation in leukemia cell lines, but there is no cell death or cytotoxicity in NIH cells (as control). The result indicated that the HALOA complex induces apoptosis in leukemia cells. The flow cytometry result showed a significant increase in apoptosis rate from 2.23% to 15.5% (p-value- 0.012), whereas in apo α-LA and OA treated cells, necrosis was higher than apoptosis. Further, the EdU assay indicated that maximum apoptosis occurs in the G2/M phase by a 73.20% reduction in the proliferation rate. In leukemia, most of the cells have been found in the G2/M phase. The G2/M phase is tightly regulated by anti-apoptotic factor survivin by interacting with the DNA repair complex [40, 41] and is also involved in the cell-cycle-regulated expression. The microtubules of the mitotic spindle fiber consist of survivin, which helps maintain high fidelity of cell cycle checkpoints and transitions. Survivin expression is up-regulated in a cell cycle-dependent manner at the G2/M phase [38]. The expression of survivin was found to be significantly high in K562 cells, and treatment with HALOA complex along with their single constituent leads to lowering the expression of survivin. Still, the HALOA complex significantly reduces the expression of survivin and triggers apoptosis in leukemia cells. The total antioxidant is a positive indicator of apoptosis and was significantly elevated in cells treated with HALOA complex compared with apo-LA and OA. Increased production of TAC lower down inflammation in the cells [27, 28]. We observed the expression level of IL-8 during treatment with the HALOA complex and found that only the complex can significantly reduce the expression level of IL-8. Il-8 increases the invasiveness and metastatic potential in leukemia cells. So, from our result, we can infer that the complex can inhibit the metastasis in myeloid leukemia cells by elevating the expression of IL-8. Furthermore, the in silico study validated our result performed by docking, which demonstrates that the complex can block the phosphorylated (Thr-34) site of survivin. The survivin expression is high in most cancers, which correlates with inhibition of apoptosis and leads to resistance towards chemotherapy and aggressiveness of cancers. The HALOA complex will be helpful in the treatment of resistant myeloid leukemia by targeting the survivin. We found a strong correlation between IL-8, survivin, and TAC using the Pearson correlation method. IL-8 and survivin were positively correlated (Pearson correlation 0.754, sig. (2-tailed) 0.246 with N=4). TAC is negatively correlated with IL-8 and survivin (Pearson correlation -0.672 and -0.481, signal (2-tailed) 0.328 and 0.519 respectively with N=4). Consequently, we can speculate that an increase in TAC level decreases the expression of IL-8, an important parameter for angiogenesis and survivin, as shown in Figure 7. The overall outcome reveals the multifaceted role of the HALOA complex with the potential to target the survivin, hampering the growth of cancer cells, increasing the TAC to counter the effect of oxidative stress, and does not allow the cell to become anoikis resistant.

## Conclusion

Overall, this study indicates the role of the HALOA complex as a new therapeutic target for drug-resistant chronic myeloid leukemia (CML) by enhancing total antioxidant activity and triggering apoptosis at the G2/M phase of cell cycle via lowering the expression level of survivin.

## Acknowledgments

### Conflict of interest

The authors declare that there is no conflict of interest.

### Authorship

V.S. and R.S. conceived and designed the research. V.S. conducted experiments. D.K. contributed new reagents or analytical tools. V.S, R.S., D.K., A.A.M, and A.K.T. analyzed the data. V.S. wrote the manuscript. All authors read and approved the manuscript, and all data were generated in-house. This study is part of the doctoral thesis of VS.

### Funding

The work was supported by the Department of Biotechnology (BT/IN/Indo-US/Foldscope/39/2015) and ICMR (45/3/2019-Hae/BMS) India.

## Supplementary Material

Supplemental data file. The above table is a mathematical extension of Figure 1, which shows the overall docking of different clusters.

**Table:** 

**S.No.**	**Cluster**	**Element**	**Full Fitness** **(Kcal/mol)**	**Estimated ΔG** **(kcal/mol)**
**1.**	0	0	-924.63	-7.80
2.	1	0	-924.00	-7.18
3.	2	0	-923.06	-6.60
4.	3	0	-922.83	-6.30

The allowed region for the protein-lipid complex formation.

Below is an extension of Figure: A – native HLALB; B – Apo-LALAB, and C – the HALOA complex and consists of the CD result which was analyzed using the Bestsel Tool.
Below is an extension of Figure 4; here we have also tested the toxicity of the HALOA complex on a normal cell line (NIH), as shown:
Below is an extension of Figure 6 (docking of survivin with the complex). The table and figure show the overall rank of docking with total energy allowed for the perfect docking.

**Table:** 

**Conf**	**Electrostatics**	**Desolvation**	**VdW**	**Total**	**Rank**
**6566**	-8.720	-23.299	66.365	-25.382	1
**8811**	-6.992	-23.756	64.671	-24.281	2
**3429**	-8.533	-24.043	84.989	-24.077	3
**1971**	-14.420	-18.187	102.243	-22.382	4
**8711**	-17.947	-8.545	44.566	-22.035	5
**5042**	-10.220	-14.999	37.852	-21.434	6
**6208**	-22.148	-3.775	44.948	-21.429	7
**2309**	-15.289	-10.353	47.372	-20.904	8
**3187**	-10.129	-17.234	73.697	-19.994	9
**7590**	-13.355	-12.108	54.836	-19.980	10

The table shows the energy prediction of docking (top 10 model) with high efficiency.

**Figure:**
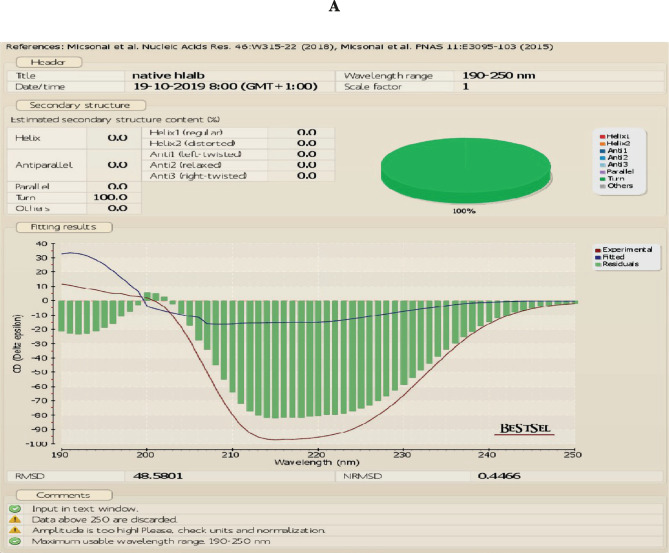


**Figure:**
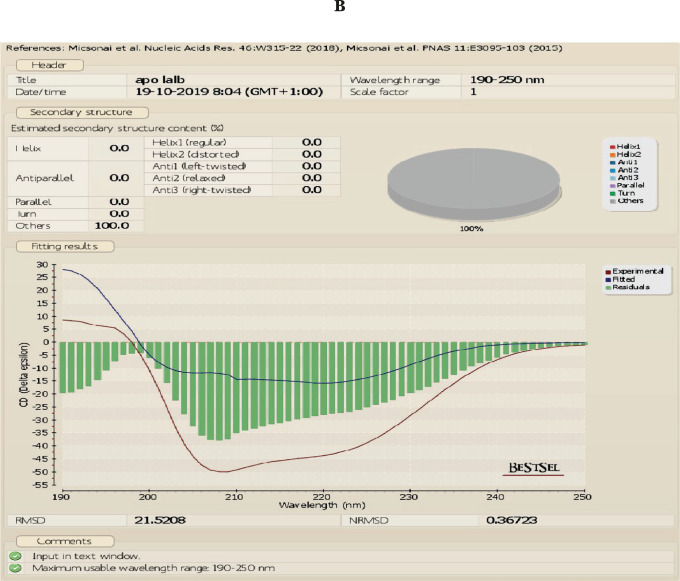


**Figure:**
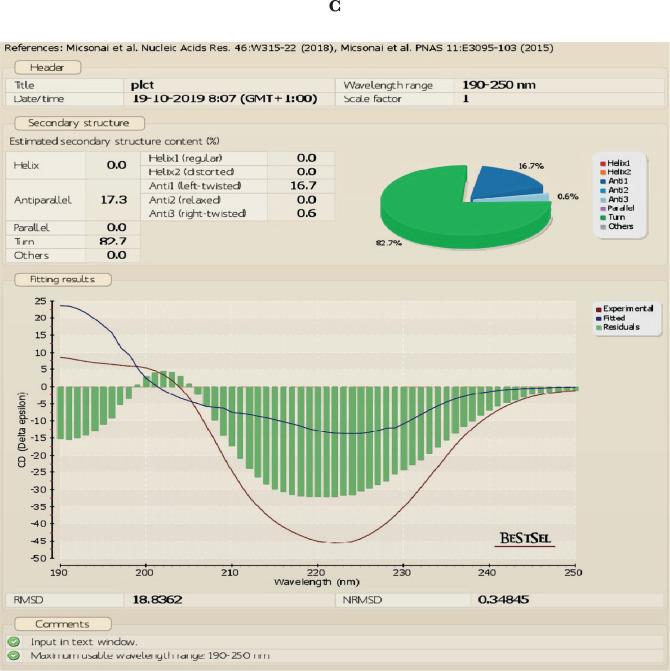


**Figure:**
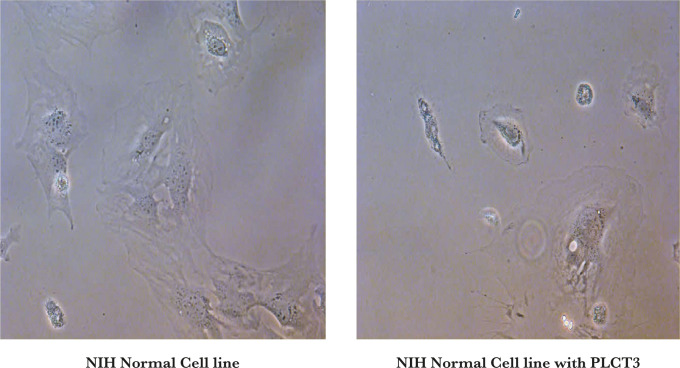


**Figure:**