# Supplementary material for: A new variant of the human α-lactalbumin-oleic acid complex as an anticancer agent for chronic myeloid leukemia
Source: J Med Life. 2021 Sep-Oct;14(5):620–35. doi: 10.25122/jml-2021-0065 (PMC8742887; doi:10.25122/jml-2021-0065)
Supplement: Supplemental data file. [file JMedLife-14-620-S.pdf]

The above table is a mathematical extension of Figure 1, which shows the overall docking of different clusters.

S.No.	Cluster	Element	Full Fitness (Kcal/mol)	Estimated ΔG (kcal/mol)
**1.**	0	0	-924.63	-7.80
**2.**	1	0	-924.00	-7.18
**3.**	2	0	-923.06	-6.60
**4.**	3	0	-922.83	-6.30

The allowed region for the protein-lipid complex formation.

Below is an extension of Figure: A – native HLALB; B – Apo-LALAB, and C – the HALOA complex and consists of the CD result which was analyzed using the Bestsel Tool.

A

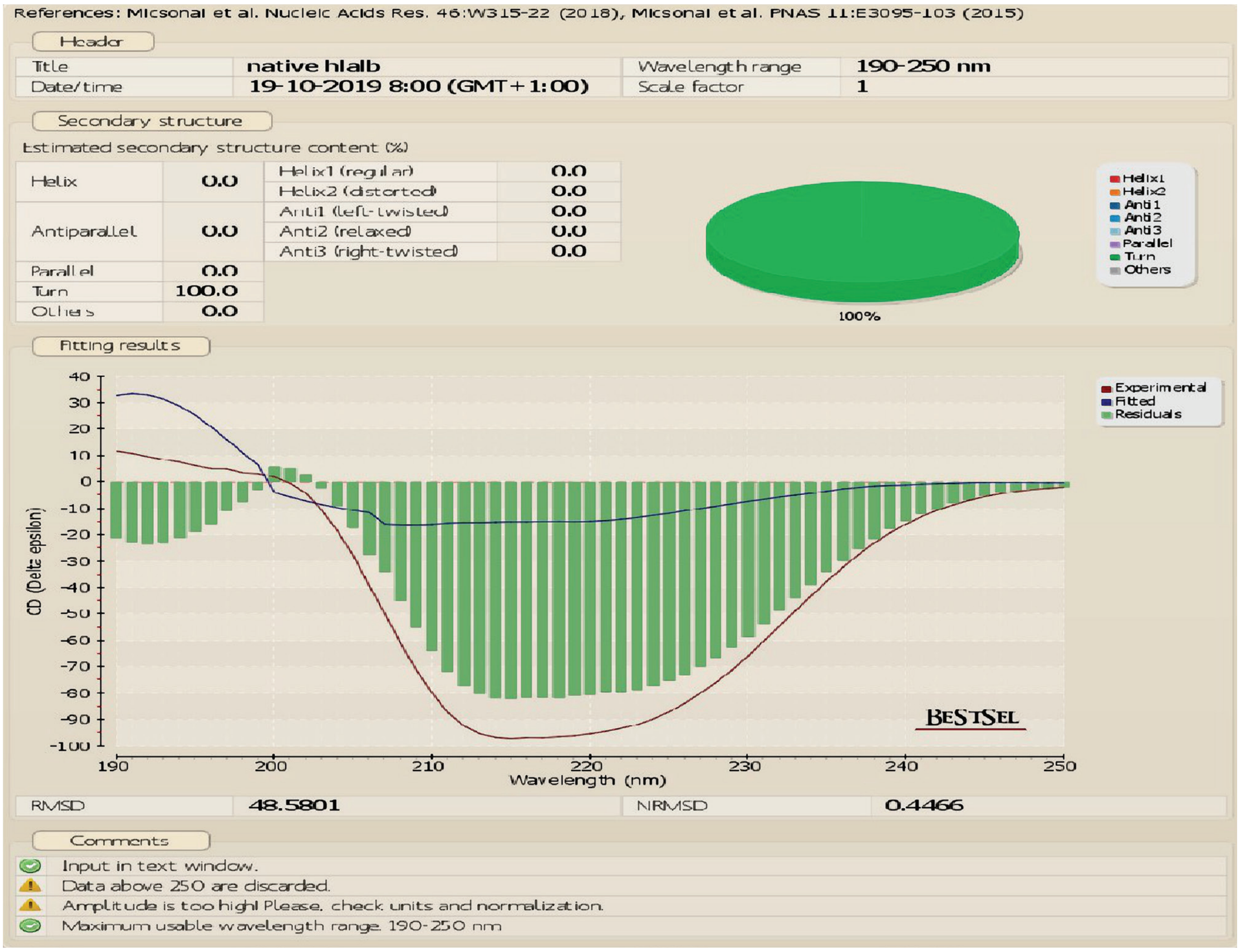

B

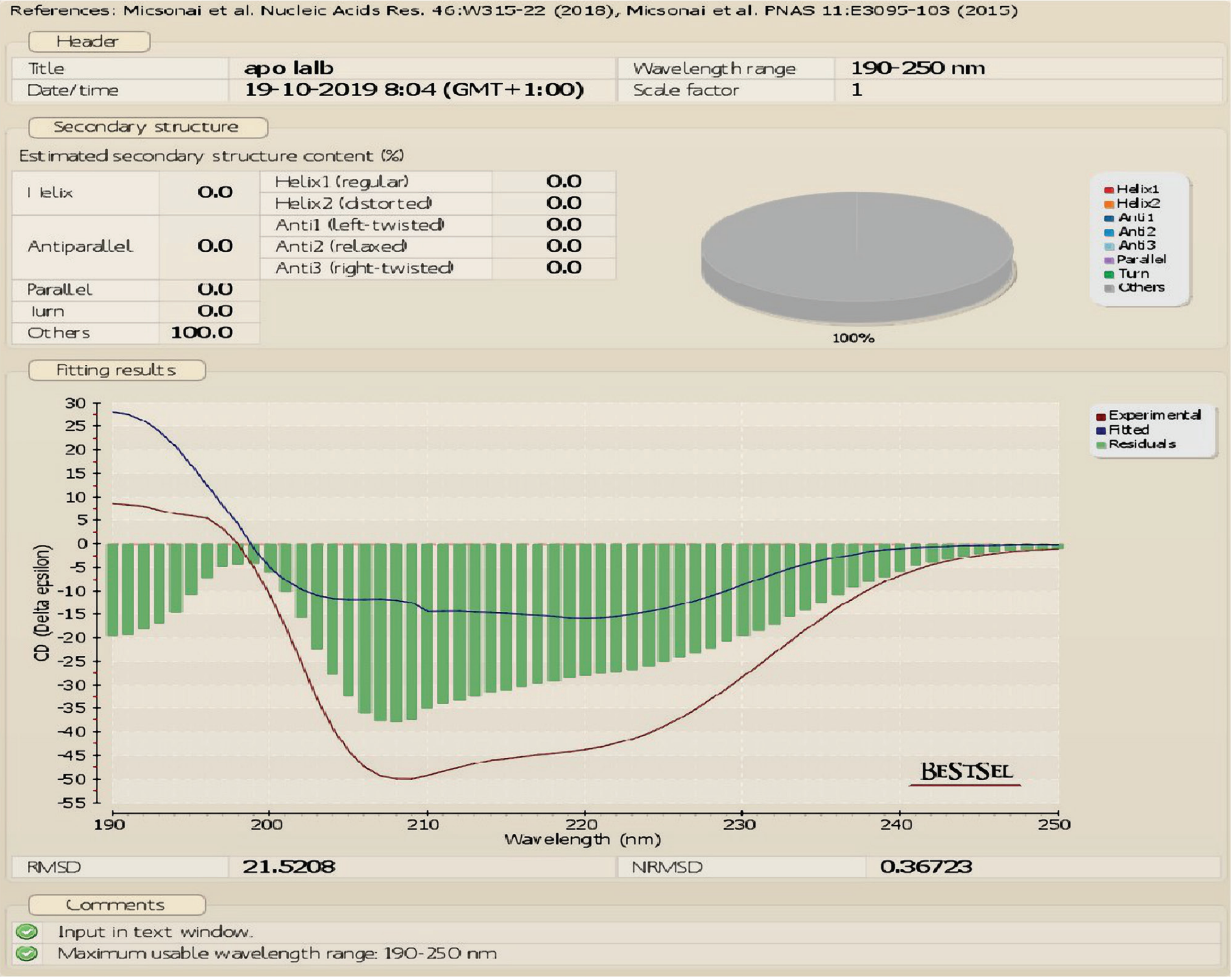
References: Micsonai et al. Nucleic Acids Res. 46:W315-22 (2018), Micsonai et al. PNAS 11:E3095-103 (2015)
Header
Title
apo lalb
Date/time
19-10-2019 8:04 (GMT+1:00)
Wavelength range
190-250 nm
Scale factor
1
Secondary structure
Estimated secondary structure content (%)
Helix 0.0
Helix1 (regular) 0.0
Helix2 (distorted) 0.0
Antiparallel 0.0
Anti1 (left-twisted) 0.0
Anti2 (relaxed) 0.0
Anti3 (right-twisted) 0.0
Parallel 0.0
Turn 0.0
Others 100.0
100%
Helix1
Helix2
Anti1
Anti2
Anti3
Parallel
Turn
Others
Fitting results
CD (Delta epsilon)
Wavelength (nm)
Experimental
Fitted
Residuals
BeStSel
RMSD
21.5208
NRMSD
0.36723
Comments
Input in text window.
Maximum usable wavelength range: 190-250 nm

C

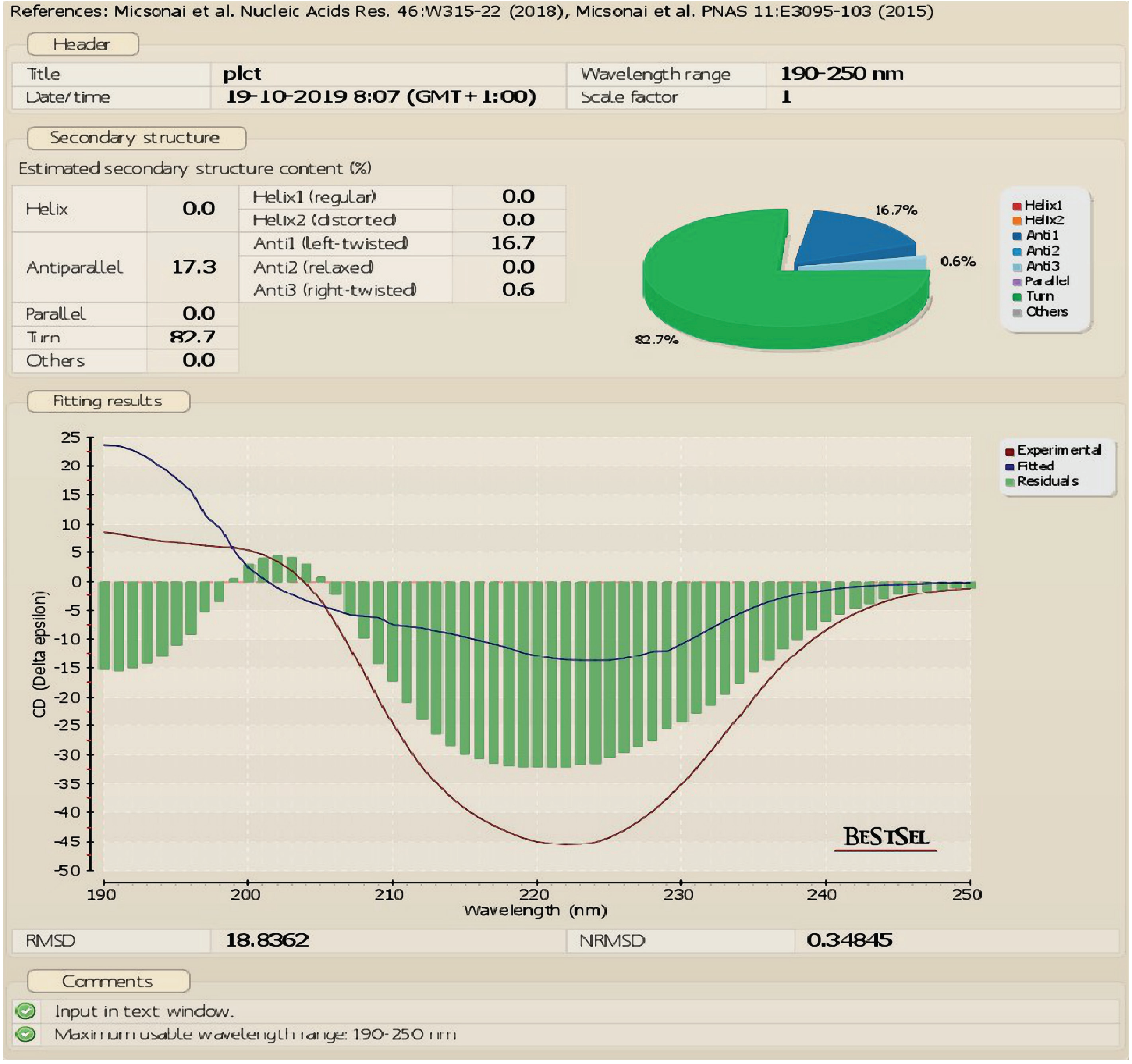
References: Micsonai et al. Nucleic Acids Res. 46:W315-22 (2018), Micsonai et al. PNAS 11:E3095-103 (2015)
Header
Title
plct
Date/time
19-10-2019 8:07 (GMT+1:00)
Wavelength range
190-250 nm
Scale factor
1
Secondary structure
Estimated secondary structure content (%)
Helix 0.0
Helix1 (regular) 0.0
Helix2 (distorted) 0.0
Antiparallel 17.3
Anti1 (left-twisted) 16.7
Anti2 (relaxed) 0.0
Anti3 (right-twisted) 0.6
Parallel 0.0
Turn 82.7
Others 0.0
16.7%
0.6%
82.7%
Helix1
Helix2
Anti1
Anti2
Anti3
Parallel
Turn
Others
Fitting results
CD (Delta epsilon)
Wavelength (nm)
Experimental
Fitted
Residuals
BeStSel
RMSD 18.8362
NRMSD 0.34845
Comments
Input in text window.
Maximum usable wavelength range: 190-250 nm

Below is an extension of Figure 4; here we have also tested the toxicity of the HALOA complex on a normal cell line (NIH), as shown:

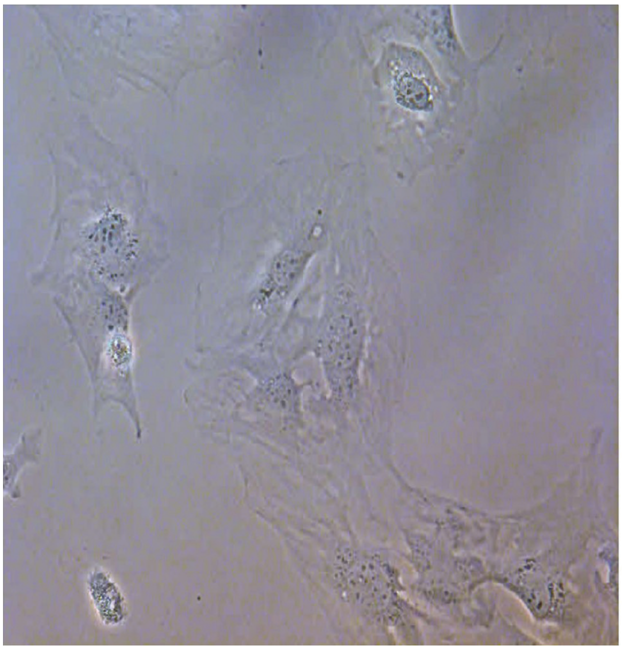

NIH Normal Cell line

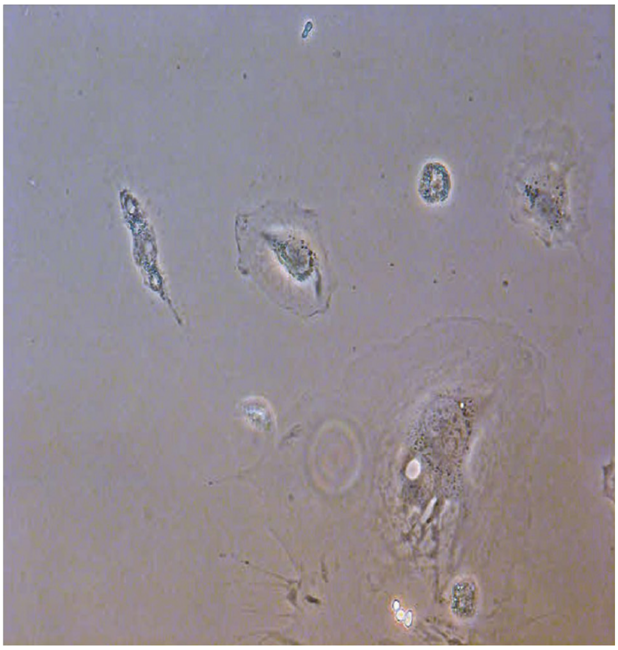

NIH Normal Cell line with PLCT3

Below is an extension of Figure 6 (docking of survivin with the complex). The table and figure show the overall rank of docking with total energy allowed for the perfect docking.

Conf	Electrostatics	Desolvation	VdW	Total	Rank
**6566**	-8.720	-23.299	66.365	-25.382	1
**8811**	-6.992	-23.756	64.671	-24.281	2
**3429**	-8.533	-24.043	84.989	-24.077	3
**1971**	-14.420	-18.187	102.243	-22.382	4
**8711**	-17.947	-8.545	44.566	-22.035	5
**5042**	-10.220	-14.999	37.852	-21.434	6
**6208**	-22.148	-3.775	44.948	-21.429	7
**2309**	-15.289	-10.353	47.372	-20.904	8
**3187**	-10.129	-17.234	73.697	-19.994	9
**7590**	-13.355	-12.108	54.836	-19.980	10

The table shows the energy prediction of docking (top 10 model) with high efficiency.

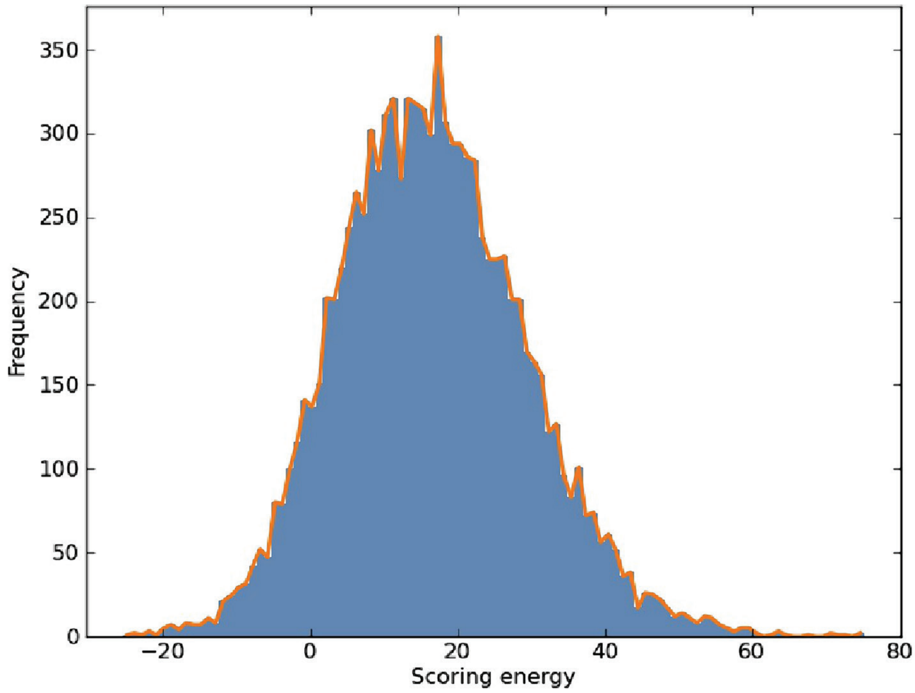

Docking energy score
